# The effect of menstrual education on knowledge on menstruation: a quasi-experimental study

**DOI:** 10.1590/1980-220X-REEUSP-2025-0459en

**Published:** 2026-03-20

**Authors:** Reyyan Gürel, Yasemin Eskigülek

**Affiliations:** 1 Republic of Türkiye Ministry of Health, General Directorate of Public Hospitals, Ankara, Türkiye.; 2 Başkent University, Faculty of Health Sciences, Department of Nursing, Ankara, Türkiye.

**Keywords:** Adolescent, Menstruation, Menstrual Cycle, Adolescente, Menstruação, Ciclo Menstrual

## Abstract

**Objective:**

This study aimed to assess the effect of menstruation education on adolescent girls’ knowledge levels.

**Method:**

A quasi-experimental single-group pre and post-test design was conducted with 267 adolescent girls from a public secondary school in Ankara. Data were collected using a sociodemographic form and a knowledge test form, and multiple linear regression was used to identify factors affecting knowledge levels.

**Results:**

Pretest knowledge scores differed significantly by age, grade, mother’s education level, menarche status, and prior menstruation information (p < 0.05). Menarche status and mother’s education accounted for 11% of the variance in pretest knowledge scores (r = 0.33).

**Conclusion:**

Menstruation education significantly increased adolescents’ knowledge levels. To support public health, school-based menstrual hygiene education delivered by healthcare professionals is recommended.

## RESUMO

**Objetivo:**

Este estudo teve como objetivo avaliar o efeito da educação sobre a menstruação nos níveis de conhecimento de adolescentes do sexo feminino.

**Método:**

Foi conduzido um estudo quase experimental com delineamento de grupo único, utilizando pré-teste e pós-teste, com a participação de 267 adolescentes de uma escola pública de ensino fundamental em Ancara. Os dados foram coletados por meio de um formulário sociodemográfico e de um teste de conhecimento, e a regressão linear múltipla foi utilizada para identificar os fatores que influenciam os níveis de conhecimento.

**Resultados:**

As pontuações de conhecimento no pré-teste diferiram significativamente de acordo com idade, série escolar, nível de escolaridade materna, estado de menarca e informações prévias sobre menstruação (p < 0.05). O estado de menarca e a escolaridade materna explicaram 11% da variância das pontuações de conhecimento no pré-teste (r = 0.33).

**Conclusão:**

A educação sobre a menstruação aumentou significativamente os níveis de conhecimento das adolescentes. Para o fortalecimento da saúde pública, recomenda-se a implementação de educação em higiene menstrual no ambiente escolar, ministrada por profissionais de saúde.

## INTRODUCTION

Menstruation is a natural physiological process that begins with menarche and continues until menopause, with an average onset age between 11 and 16 years, depending on individual and environmental factors^(1)^. This period coincides with adolescence, which the World Health Organization defines as a critical developmental stage characterized by rapid physical, sexual, and psychosocial changes^(2,3)^. Adolescents constitute approximately one-fifth of the global population of nearly eight billion^(4)^. In Türkiye, 15.2% of the total population is young, and 29.7% of this group consists of adolescents^(5)^. The onset of menarche is considered a significant milestone for adolescent girls during this transitional phase^(6)^.

Despite being a normal biological process, menstruation is often surrounded by myths and sociocultural taboos, negatively influencing adolescents’ perceptions and experiences^(7)^. Studies have shown that many adolescent girls lack sufficient knowledge about menstruation and are exposed to misinformation^(7,8,9,10,11,12,13)^. Common misconceptions include dietary restrictions, avoidance of bathing, changing clothes, combing hair, or entering sacred places during menstruation^(11)^, as well as practices such as hiding or burying menstrual products due to beliefs that menstruating women are impure or shameful^(9)^. These beliefs may cause menstruation to be perceived as embarrassing, frightening, and confusing, potentially leading to adverse physical and psychological health outcomes.

Qualitative and quantitative studies have reported that adolescent girls frequently experience intense fear and anxiety at the time of menarche and often refrain from discussing their experiences with others^(10,14)^. In addition to emotional challenges, inadequate menstrual hygiene practices remain a significant concern. Previous research has demonstrated insufficient knowledge regarding menstrual hygiene among adolescent girls, including appropriate frequency of changing sanitary pads and maintaining toilet hygiene^(8,15)^. Providing accurate and age-appropriate information about menstruation facilitates adolescents’ adaptation to the menstrual cycle and promotes healthier behaviors^(1)^.

Schools provide a suitable and accessible environment for delivering menstruation education, as they allow for continuous monitoring, guidance, and early intervention during adolescence. Given the difficulties adolescent girls face in adapting to the physical and psychological changes associated with menstruation^(6)^, raising awareness about menstrual-related changes and effective coping strategies is essential. Early and structured education plays a critical role in shaping positive attitudes toward menstruation, dispelling myths and taboos, improving hygiene practices, and enabling adolescent girls to manage menstruation with confidence. By identifying knowledge gaps and emphasizing the importance of age-appropriate education, the present study aims to contribute to the existing literature and inform the development of effective menstrual health interventions for adolescent girls. This study differs from previous research owing to its potential to improve public health outcomes and promote educational equity. Conducted in a public secondary school located in a socioeconomically disadvantaged area, it targeted a population of adolescents at higher risk of limited access to accurate reproductive health information. By identifying knowledge gaps and emphasizing the importance of age-appropriate education, the study aimed to evaluate the effectiveness of menstruation education in improving menstrual knowledge among adolescent girls with greater educational needs and potential public health impact, while contributing to the existing literature and informing the development of effective menstrual health interventions.

Based on the above discussion and the literature, we formulated the following hypothesis: H1: Menstruation education given to adolescent girls affects their knowledge of menstruation. H1 was formulated as non-directional (two-tailed) hypothesis.

## Method

### Design of Study

This study was conducted using single-group pre and post-test quasi-experimental design, and due to the nature of the intervention, no blinding was applied to either the adolescent girls or the researchers. The study used the Transparent Reporting of Evaluations with Nonrandomized Designs (TREND) statement, which enables the transparent reporting of nonrandomized controlled studies to establish an evidence base for making decisions related to public health^(16)^.

### Sample Size Calculation

**Figure 1:**
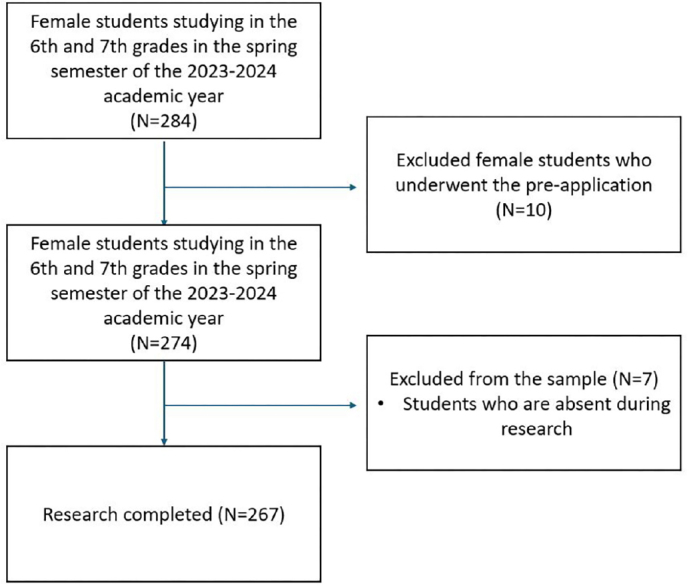
Flow diagram of the study.

The study population consisted of all 284 adolescent girls enrolled in the 6th and 7th grades of a secondary school in Ankara city center, during spring semester of the 2023-2024 academic year. Since it was stated in the literature that the average age of menarche in Türkiye was 12-13 years, the population consisted of 6^th^ and 7^th^ grade^(17)^. The sample size was calculated as 199 students with 80% power, an alpha error probability of 0.05, and a small effect size (Cohen d = 0.2) using a two-tailed distribution. The study was completed with 267 adolescent girls, achieving 90% power. In single-group pre- and post-test designs, an effect size of 0.2 is commonly recommended for detecting small but meaningful changes; therefore, this cutoff was chosen for the sample size calculation^(18)^. The G Power 3.1.9.4 program was used to calculate the sample size. Convenience sampling method was used to conduct the study. At the time of the study, 10 adolescent girls who reported receiving prior education on menstruation from their teachers and 7 adolescent girls who did not attend the education during the study were excluded from the sample. The sample consisted of 267 adolescent girls who were present at the school during the data collection and answered the data collection forms before the education (BE) and one month after the education (AE). A flow diagram was prepared based on the TREND checklist^(16)^ and Figure 1 shows the flow diagram of the study.

### Inclusion and Exclusion Criteria

The inclusion criteria were as follows: having biological female characteristic; being between the ages of 11 and 14; being enrolled in the 6th or 7th grade of school where the education is provided; being at school on the day of the study; having no barriers to communication; having no barriers to answering the pretest and post-test questions; volunteering to participate in the education.

The exclusion criteria were as follows: being unable to participate in education due to special educational needs; not participating in the education to be given within the scope of the research; previously receiving formal education about menstruation.

### Data Collection Tools

The research data were collected from adolescent girls enrolled in the 6th and 7th grades at a secondary school located in the city center of Ankara during the spring semester of the 2023-2024 academic year, using a “Socio-Demographic Form” and a “Knowledge Test Form” administered through face-to-face interviews.

The socio-demographic form created by researchers consists of a total of nine questions: six questions covering sociodemographic characteristics such as the adolescent girl’s age, grade level, mother’s educational status, father’s educational status, mother’s employment status, and father’s employment status; and three questions covering menstrual characteristics, such as menarche, knowledge of information related to menstruation, and the source of information received about menstruation^(1,11)^.

The knowledge test form consisted of 10 questions about the menstrual period and hygiene practices related to menstruation. In addition, the form contained questions about the adolescent girls’ name, surname, and grade level. The questions had “true”, “false” and “I don’t know” options. To evaluate the content validity of a scale, it is recommended to obtain feedback from 3 to 20 experts regarding the appropriateness of the scale items^(19)^. Therefore, expert opinions were sought from the school counselor and three academics to ensure the relevance and suitability of the questions in the knowledge test. The content validity index for each question in the form, calculated using the Davis method, ranged between 0.8 and 1.0.^(20)^. Menstruation knowledge test was used for both pretest and post-test data collection. To evaluate the comprehensibility of the data collection forms, a pre-application was conducted with 10 adolescent girls out of the sample. After the pre-application, the data collection forms were finalized. The internal consistency of the knowledge test was examined using the Kuder–Richardson Formula 20 (KR-20). For the pretest administration, the KR-20 coefficient was calculated as 0.48, indicating a relatively low internal consistency. In the post-test, the KR-20 coefficient further decreased to 0.26. This decrease can be explained by the reduced variance of total scores in the post-test, as the majority of adolescent girls achieved very high scores (M = 9.63, SD = 0.88). These findings suggest that while the test was useful in detecting differences in baseline knowledge, it became less discriminative after the intervention, when adolescent girls’ knowledge levels were homogenously high.

### Data Collection

Prior to the intervention, adolescent girls’ baseline knowledge about menstruation was assessed. The educational program aimed to enhance their understanding and included a PowerPoint presentation covering the anatomy of female internal and external genital organs, the physiology of menstruation, menstrual and perineal hygiene, common myths about menstruation, and psychosocial support. The sessions were delivered in person using interactive techniques, including question-and-answer, demonstration, and group discussion. During demonstrations, researchers first explained and modeled menstrual hygiene practices, after which adolescent girls practiced these activities themselves. To encourage engagement, correct responses during Q&A segments were rewarded with pens. Adolescent girls were organized into groups of approximately 50 girls per session, and all sessions were conducted in the school’s conference hall. The intervention comprised two approximately 40-minute sessions, resulting in a total duration of 80 minutes. The study process began with the collection of adolescent girls’ socio-demographic form, followed by the administration of a Knowledge Test Form as the pretest. The educational sessions were then conducted, and one month after the intervention, the same Knowledge Test Form was readministered as the post-test. Prior to the sessions, adolescent girls were informed about the purpose of the education, the forms to be completed, and how to fill them out, which, along with the interactive incentives, served to enhance adherence and participation.

### Outcomes

The primary outcome of the study was the change in the total menstruation knowledge score of adolescent girls before and after the education. The secondary outcome was the difference in post-intervention knowledge levels according to the menarche status of the adolescent girls.

### Data Analysis and Treatment

The data of the study were analyzed with Statistical Package for the Social Sciences (SPSS) version 25.0. Categorical data were expressed as percentage, mean and standard deviation. The normal distribution of data was tested with Kolmogorov-Smirnov. Paired sample t test and Pearson correlation analysis were used to compare pretest and post-test scores of knowledge test. Multiple linear regression analysis was performed to determine the factors affecting knowledge level about menstruation. Cohen’s d coefficient was used to calculate the effect size^(18)^. The statistical significance level of 0.05 was accepted. One-way analysis of variance (ANOVA) was used to compare the scores of three or more groups, and Bonferroni post hoc test was used to determine the different group. Multiple linear regression analysis was used to determine the relationship between dependent and independent variables. After the normality assumption was met for the analysis, the following variables were added using the stepwise method: age of student, grade of education, educational level and employment status of mother and father, menarche status, receiving information about menstruation and source of information about menstruation. The regression analysis conditions were met by determining that the model did not have multicollinearity (Variance Inflation Factor: 1.004) and autocorrelation (Durbin-Watson: 1.857) problems and that the standard residuals were between (-3+3) at 95% confidence interval^(21)^.

### Ethical Aspects

Research permission was obtained from Başkent University Social and Human Sciences Scientific Research and Publication Ethics Committee (Date:6.11.2023-Number: 17162298.600-241). Following the approval of the ethics committee, institutional permission was also obtained from the school where the study would be conducted. A written statement explaining the purpose of the study was included in the introduction part of the data collection form and adolescent girls agreeing to participate in the study after reading it were included in the study. In addition, the data set containing the answers filled in by the adolescent girls was saved in an encrypted file on the computer, and after the data analysis was completed, the data was deleted to ensure the confidentiality of personal data. The study was conducted in accordance with the “Declaration of Helsinki”.

## RESULTS

The comparison of adolescent girls’ pretest and post-test knowledge scores on menstruation according to socio-demographic characteristics is presented in Table 1. The mean age of the adolescent girls was 12.38 ± 0.7 years. Of the adolescent girls included in the study, 44.6% were 13 years old and 68.5% were enrolled in the seventh grade. Regarding parental education, 32.6% of mothers had completed primary school, while 40.4% of fathers were high school graduates. Most mothers were not employed (77.2%), whereas the majority of fathers were employed (93.6%). Additionally, 61.8% of the adolescent girls reported having experienced menarche, and among those who had received information about menstruation, the primary source of information was their mother (71.1%) (Table 1).

**Table 1:** Distribution of pretest and post-test knowledge mean scores of adolescent girls by sociodemographic and menstrual characteristics – Ankara, Türkiye, 2023.

Characteristics (n:267)		n	%	Pretest score	Post-test score
				Mean ± SD	Mean ± SD
**A–ge** [X̄: 12.38; SD: 0.7 years]	11	32	12.0	6.03 ± 1.7	8.87 ± 1.1
	12	107	40.1	5.97 ± 2.1	9.26 ± 1.2
	13	119	44.6	6.76 ± 1.7	9.14 ± 1.3
	14	9	3.4	6.55 ± 1.7	9.00 ± 0.9
Test				^†^3.798; ^*^0.005 ^a^	^†^0.798; ^*^0.527
**Grade**	6	84	31.5	6.01 ± 2.0	9.04 ± 1.3
	7	183	68.5	6.53 ± 1.9	9.20 ± 1.2
Test				^‡^–2.085; ^*^0.038	^‡^–1.006; ^*^0.315
**Educational level of mother**	Illiterate	5	1.9	4.80 ± 2.2	7.20 ± 2.9
	Primary school	87	32.6	6.06 ± 1.9	9.21 ± 1.1
	Secondary school	78	29.2	6.33 ± 1.8	9.01 ± 1.3
	High school	82	30.7	6.68 ± 1.9	9.30 ± 1.1
	University	15	5.6	7.06 ± 1.5	9.40 ± 0.8
Test				^†^2.529; ^*^0.041 ^b^	^†^4.283; ^*^0.002 ^b^
**Educational level of father**	Illiterate	5	1.9	5.40 ± 2.4	9.40 ± 0.9
	Primary school	44	16.5	6.00 ± 1.9	9.22 ± 0.9
	Secondary school	81	30.3	6.17 ± 1.8	9.03 ± 1.5
	High school	108	40.4	6.69 ± 1.9	9.15 ± 1.2
	University	29	10.9	6.41 ± 2.1	9.34 ± 0.7
Test				^†^1.777; ^*^0.134	^†^0.460; ^*^0.765
**Employment status of mother**	Employed	61	22.8	6.40 ± 1.8	9.09 ± 1.3
	Not employed	206	77.2	6.35 ± 1.9	9.17 ± 1.2
Test				^‡^ 0.200; ^*^0.842	^‡^ –0.434; ^*^ 0.665
**Employment status of father**	Employed	250	93.6	6.38 ± 1.9	9.18 ± 1.2
	Not employed	17	6.4	6.05 ± 1.7	8.70 ± 1.7
Test				^‡^0.691; ^*^0.490	^‡^1.599; ^*^0.111
**Menarche**	Yes	165	61.8	6.78 ± 1.7	9.22 ± 0.1
	No	102	38.2	5.69 ± 2.0	9.04 ± 0.1
Test				^‡^4.795; ^*^0.001	^‡^1.153; ^*^0.250
**Source of information on menstruation**	Mother	165	71.1	6.60 ± 1.75	9.20 ± 1.20
	Friend or sibling	65	28.9	6.16 ± 1.84	8.98 ± 1.34
Test				^‡^1.678; ^*^0.095	^‡^1.185; ^*^0.237
**Receiving information about menstruation**	Yes	230	86.1	6.50 ± 1.8	9.20 ± 1.2
	No	37	13.9	5.48 ± 2.5	8.94 ± 1.2
Test				^‡^2.433; ^*^0.019	^‡^0.925; ^*^0.252

^a^According to Bonferroni test, difference is between 12 and 13 years old;

^b^According to Bonferroni test, difference stems from the group with illiterate mothers;

^†^: One-way analysis of variance;

^‡^: Student t test; SD: Standard Deviation; X̄: Mean;

^*^p < 0.05.

A significant difference was observed in pretest knowledge scores according to age (p = 0.005), with 12-year-old girls demonstrating lower mean scores than 13-year-old girls (5.97 ± 2.1 vs. 6.76 ± 1.7, respectively). Similarly, seventh-grade students had significantly higher pretest knowledge scores compared with sixth-grade students (p = 0.038).

Maternal education level was found to be significantly associated with both pretest (p = 0.041) and post-test (p = 0.002) knowledge scores. Adolescent girls whose mothers were illiterate had lower mean scores compared with those whose mothers had any level of formal education. The mean pretest and post-test knowledge scores were 4.80 ± 2.2 and 7.20 ± 2.9 for girls with illiterate mothers; 6.06 ± 1.9 and 9.21 ± 1.1 for those whose mothers were primary school graduates; 6.33 ± 1.8 and 9.01 ± 1.3 for secondary school graduates; 6.68 ± 1.9 and 9.30 ± 1.1 for high school graduates; and 7.06 ± 1.5 and 9.40 ± 0.8 for university graduates, respectively. In contrast, paternal education level and the employment status of mothers and fathers were not associated with significant differences in pretest or post-test knowledge scores (p > 0.05) (Table 1).

The comparison of knowledge scores according to menstruation-related characteristics is also shown in Table 1. Pretest knowledge scores were significantly higher among girls who had experienced menarche compared with those who had not (p = 0.001), whereas post-test scores did not differ significantly between the two groups (p = 0.250). Similarly, adolescent girls who had previously received information about menstruation demonstrated higher pretest knowledge scores than those who had not (p = 0.019); however, post-test knowledge scores were comparable between the groups (p = 0.252).

Table 2 presents the overall comparison of pretest and post-test knowledge scores. The mean pretest knowledge score of all participants was 7.16 ± 1.8, which increased to 9.62 ± 0.8 after the intervention. This increase was statistically significant (p = 0.001), with a Cohen’s d effect size of 1.3, indicating a strong effect. When analyzed by grade level, the mean pretest and post-test scores were 6.85 ± 1.9 and 9.54 ± 0.7 for sixth-grade students, and 7.30 ± 1.7 and 9.66 ± 0.9 for seventh-grade students, respectively. The corresponding Cohen’s d values were 1.7 for sixth-grade and 1.4 for seventh-grade students, reflecting strong effect sizes (Table 2).

**Table 2:** Comparison of pretest and post-test knowledge mean scores of adolescent girls – Ankara, Türkiye, 2023.

Tests	n	X̄ ± SD	Correlation		Paired samples t test			Cohen’s d
			r	p	t	SD	p	
**Pretest**	267	7.16 ± 1.84	^§^0.16	^*^0.007	^||^–21.08	266	^*^0.001	1.35
**Post-test**	267	9.62 ± 0.87
**6^th^grade pretest**	84	6.85 ± 1.98	^§^0.12	^*^0.275	^||^–12.04	83	^*^0.001	1.78
**6^th^grade post-test**	84	9.54 ± 0.78
**7^th^grade pretest**	183	7.30 ± 1.76	^§^0.17	^*^0.017	^||^–17.35	182	^*^0.001	1.40
**7^th^grade post-test**	183	9.66 ± 0.91						

§: Pearson correlation coefficient;

^||^: Paired samples t test; SD: Standard Deviation; X̄ Mean;

^*^p < 0.05.

The multiple linear regression analysis performed to determine the independent variables affecting the knowledge level of menstruation was shown in Table 3. The analysis was performed for the pretest knowledge mean score. Menarche status and maternal education level emerged as statistically significant predictors (p = 0.001). Together, these variables explained 11% of the variance in pretest knowledge scores, and a moderate positive correlation was observed between these factors and pretest knowledge scores (r = 0.33).

**Table 3:** Multiple linear regression analysis of variables affecting menstrual knowledge level – Ankara, Türkiye, 2023.

Model	b	SE	β	t (p)	R	R2
**Menarche**	–1.062	0.227	–0.272	–4.674 (**0.001**)	0.33	0.11
**Educational level of mother**	0.324	0.115	0.165	2.825 (0.005)		

β: Standardized beta coefficients; b: Unstandardized regression coefficient; R: Multiple correlation coefficient; SE: Standard Error.

## DISCUSSION

This study examined adolescent girls’ knowledge and practices related to menstruation and evaluated the effectiveness of a structured educational intervention. Although the age of menarche varies depending on biological, environmental, and sociocultural factors, it generally occurs between 11 and 16 years of age^(1)^, with an average age of 13 years reported in the literature^(17,22)^. In the present study, seventh-grade adolescent girls, most of whom were 13 years old, demonstrated higher pretest knowledge scores regarding menstruation compared to other age groups. This finding is likely associated with increased awareness, early personal experience with menstruation^(23)^, and heightened interest in menstrual issues during this developmental period^(24)^.

Previous studies have reported that adolescents’ knowledge of menstrual hygiene management is often inadequate and influenced by cultural taboos^(14)^. However, some studies indicate that a considerable proportion of girls possess sufficient menstrual knowledge prior to menarche^(6)^. In the present study, higher pretest knowledge levels among 13-year-old girls and those who had already experienced menarche may be explained by personal experience and informal learning through family and social environments. Differences observed across studies may reflect variations in cultural norms, socioeconomic status, and access to accurate information.

Menarche commonly occurs during the seventh grade, and in this study, post-test knowledge scores of seventh-grade students were higher than those of sixth-grade students. These findings support the effectiveness of menstrual hygiene education. Similar results have been reported in previous studies, showing positive changes in bathing habits and menstrual hygiene behaviors following genital hygiene education^(25,26)^. Sinop Gedik and Şahin^(27)^ also demonstrated that menstrual hygiene education significantly improved adolescent girls’ knowledge and hygiene behaviors. Consistent with these findings, the increased post-test scores observed in this study suggest a reduction in misconceptions and an increase in awareness, leading to the acceptance of hypothesis H1. Overall, the educational intervention was effective in enhancing adolescents’ menstrual knowledge.

Accurate and reliable information about menstruation is essential for maintaining proper hygiene and managing menstruation effectively. The dissemination of menstrual health information has become increasingly important, particularly in developing countries^(28)^. Studies indicate that girls’ menstrual knowledge is frequently insufficient, and it is negatively influenced by cultural beliefs, while communication with mothers about menstruation may be limited^(11)^. The literature consistently identifies mothers as the primary source of menstrual information for girls^(6,28)^, and emphasizes that mothers’ educational level plays a critical role in ensuring accurate knowledge and healthy hygiene practices^(15)^.

In the present study, most adolescent girls identified their mothers as their main source of menstrual information, and higher maternal education levels were associated with increased menstrual knowledge among daughters. Furthermore, girls whose mothers were illiterate had lower pretest knowledge scores, highlighting the influence of maternal education on menstrual awareness. These findings are consistent with those reported by Siabani, Charehjow, and Babakhani^(29)^, who found a significant relationship between mothers’ education levels and adolescent girls’ menstrual practices. Inadequate maternal knowledge may hinder effective mother–daughter communication regarding menstrual health^(11)^, whereas improving mothers’ educational status may enhance the accuracy of information shared with their daughters^(30)^.

Menstrual awareness before menarche is recognized as a key indicator for monitoring menstrual hygiene management at both national and global levels^(11,23)^. In this study, adolescents who had experienced menarche and received information about menstruation demonstrated higher pre-education knowledge scores. While some studies report that pre-menarche girls possess limited understanding of menstruation and are unaware of the source of menstrual bleeding^(31)^, others indicate that girls who experience menarche gain greater knowledge, confidence, and competence in menstrual management^(23)^. Although Gümüş Sarı^(32)^ reported lower pre-education knowledge scores among girls who had experienced menarche, this difference was not statistically significant. The higher knowledge levels observed among post-menarche adolescents in the present study are likely attributable to personal experience and environmental learning, while discrepancies across studies may result from cultural, socioeconomic, and informational differences.

## LIMITATIONS

The limitations of this study include the inclusion of both menarche and non-menarche adolescent girls in the sample group, the lack of sufficient validity and reliability evidence for the questionnaire administered to the adolescents before and after the educational intervention, and the limited extent of expert consultation during the development of the data collection tools. In particular, the relatively low reliability of the knowledge test used in the study constitutes a clear methodological limitation and should be considered when interpreting the findings.

In addition, although a sufficient number of adolescent girls were included in this study, conducting similar studies in different regions is important for supporting accurate knowledge of the menstrual cycle, as information and perceptions related to menstruation may be influenced by cultural and environmental differences.

## CONCLUSION

In our study, it was found that education was effective, and the knowledge test scores of the adolescent girls about menstruation increased after the education. Menarche and mother’s education level were found to be factors affecting the level of knowledge about menstruation. Therefore, it is postulated that increasing the educational status of mothers will have a positive effect, increasing the correct practices related to menstruation before menarche in adolescent girls.

Findings related to factors affecting adolescent girls’ menstrual hygiene can guide policy. Policy makers may use this information to ensure that menstrual hygiene education is provided regularly in every school and included in the curriculum. It is recommended that this educational program be conducted in collaboration with specialized health personnel, and that the age and culture of adolescent girls and the myths that exist in their communities should be included in the planning of the education. In addition, this study may guide further research to investigate the effectiveness of these interventions and to identify other deficiencies in menstrual hygiene that may lead to targeted improvements.

Educating adolescent girls about menstrual hygiene is essential for promoting school health, as it equips them with the knowledge and skills to manage menstruation in a safe and hygienic manner. Proper menstrual hygiene education helps to prevent health issues, such as infections, by encouraging the use of appropriate menstrual products. Furthermore, it plays a crucial role in reducing the stigma and embarrassment often associated with menstruation, thereby fostering a supportive and inclusive environment. By incorporating menstrual hygiene education into school curricula, absenteeism may be reduced, ensuring continued participation in education and improving adolescent girls’ overall well-being.

## Data Availability

The entire dataset supporting the results of this study is available upon request to the corresponding author.
