# Supplementary material for: The effect of menstrual education on knowledge on menstruation: a quasi-experimental study
Source: Rev Esc Enferm USP. 2026 Mar 20;60:e20250459. doi: 10.1590/1980-220X-REEUSP-2025-0459en (PMC13074480; doi:10.1590/1980-220X-REEUSP-2025-0459en)
Supplement: Translation

# Translation: El efecto de la educación menstrual sobre el conocimiento acerca de la menstruación: un estudio cuasi-experimental

**DOI:** 10.1590/1980-220X-REEUSP-2025-0459es

**Authors:** Reyyan Gürel, Yasemin Eskigülek

**Affiliations:** 1 Republic of Türkiye Ministry of Health, General Directorate of Public Hospitals, Ankara, Türkiye.; 2 Başkent University, Faculty of Health Sciences, Department of Nursing, Ankara, Türkiye.

**Keywords:** Adolescente, Menstruación, Ciclo Menstrual

## Abstract

**Objetivo:**

Este estudio tuvo como objetivo evaluar el efecto de la educación sobre la menstruación en los niveles de conocimiento de adolescentes.

**Método:**

Se llevó a cabo un estudio cuasi experimental con un diseño de un solo grupo con pre test y post test, en el que participaron 267 adolescentes de una escuela secundaria pública en Ankara. Los datos se recopilaron mediante un formulario sociodemográfico y un cuestionario de conocimientos, y se utilizó regresión lineal múltiple para identificar los factores que influyen en los niveles de conocimiento.

**Resultados:**

Las puntuaciones de conocimiento en la preprueba mostraron diferencias significativas según la edad, el grado escolar, el nivel educativo de la madre, el estado de menarquia y la información previa sobre la menstruación (p < 0.05). El estado de menarquia y el nivel educativo materno explicaron el 11% de la varianza de las puntuaciones de conocimiento en el pre test (r = 0.33).

**Conclusión:**

La educación sobre la menstruación aumentó significativamente los niveles de conocimiento de las adolescentes. Para apoyar la salud pública, se recomienda la implementación de educación en higiene menstrual en el entorno escolar, impartida por profesionales de la salud.

## INTRODUCCIÓN

La menstruación es un proceso fisiológico natural que comienza con la menarca y continúa hasta la menopausia, con una edad media de inicio entre los 11 y los 16 años, dependiendo de factores individuales y ambientales^(1)^. Este periodo coincide con la adolescencia, que la Organización Mundial de la Salud define como una etapa crítica del desarrollo caracterizada por rápidos cambios físicos, sexuales y psicosociales^(2,3)^. Los ado-lescentes constituyen aproximadamente una quinta parte de la población mundial, que asciende a casi ocho mil millones de personas^(4)^. En Turquía, el 15,2% de la población total es joven, y el 29,7% de este grupo está compuesto por adolescentes^(5)^. El inicio de la menarca se considera un hito importante para las adolescentes durante esta fase de transición^(6)^.

A pesar de ser un proceso biológico normal, la menstruación suele estar rodeada de mitos y tabúes socioculturales, lo que influye negativamente en las percepciones y experiencias de las adolescentes^(7)^. Los estudios han demostrado que muchas ado-lescentes carecen de conocimientos suficientes sobre la menstruación y están expuestas a información errónea^(7,8,9,10,11,12,13)^. Entre los conceptos erróneos más comunes se encuentran las restricciones alimentarias, evitar bañarse, cambiarse de ropa, peinarse o entrar en lugares sagrados durante la menstruación^(11)^, así como prác-ticas como ocultar o enterrar los productos menstruales debido a la creencia de que las mujeres que menstrúan son impuras o vergonzosas^(9)^. Estas creencias pueden hacer que la menstruación se perciba como algo vergonzoso, aterrador y confuso, lo que puede dar lugar a consecuencias adversas para la salud física y psicológica.

Estudios cualitativos y cuantitativos han revelado que las adolescentes suelen experimentar un miedo y una ansiedad intensos en el momento de la menarca y, a menudo, se abs-tienen de hablar de sus experiencias con otras personas^(10,14)^. Además de los retos emocionales, las prácticas inadecuadas de higiene menstrual siguen siendo una preocupación importante. Investigaciones anteriores han demostrado que las adolescentes tienen un conocimiento insuficiente sobre la higiene menstrual, incluida la frecuencia adecuada para cambiar las compresas higiénicas y mantener la higiene en el baño^(8,15)^. Proporcionar información precisa y adecuada a la edad sobre la menstruación facilita la adaptación de las adolescentes al ciclo menstrual y promueve comportamientos más saludables^(1)^.

Las escuelas ofrecen un entorno adecuado y accesible para impartir educación sobre la menstruación, ya que permiten monitorear de manera continua, brindar orientación y realizar intervención temprana durante la adolescencia. Dadas las dificultades que enfrentan las adolescentes para adaptarse a los cambios físicos y psicológicos asociados con la menstruación^(6)^, es esencial crear conciencia sobre los cambios relacionados con la menstruación y las estrategias efectivas para afrontarlos. La educación temprana y estructurada desempeña un papel fundamental a la hora de fomentar actitudes positivas hacia la menstruación, disipar mitos y tabúes, mejorar las prácticas de higiene y permitir que las adolescentes gestionen la menstruación con confianza. Al identificar las lagunas de conocimiento y hacer hincapié en la importancia de una educación adecuada a la edad, el presente estudio pretende contribuir a la bibliografía existente e informar sobre el desarrollo de intervenciones eficaces en materia de salud menstrual para las adolescentes. Este estudio se diferencia de investigaciones anteriores por su potencial para mejorar los resultados de salud pública y promover la equidad en salud. Realizado en una escuela secundaria pública situada en una zona socioeconómicamente desfavorecida, se dirigió a una población de adolescentes con mayor riesgo de tener un acceso limitado a información precisa sobre salud reproductiva. Al identificar las lagunas de conocimiento y hacer hincapié en la importancia de una educación adecuada a la edad, el estudio tenía como objetivo evaluar la eficacia de la educación menstrual para mejorar los conocimientos sobre la menstruación entre las adolescentes con mayores necesidades educativas y un mayor impacto potencial en la salud pública, al tiempo que contribuía a la bibliografía existente y servía de base para el desarrollo de intervenciones eficaces en materia de salud menstrual.

Basándonos en lo anterior y en la bibliografía, formulamos la siguiente hipótesis: H1: La educación menstrual impartida a las adolescentes influye en sus conocimientos sobre la menstrua-ción. La hipótesis H1 se formuló como hipótesis no direccional (bilateral).

## MÉTODO

### Diseño Del Estudio

Este estudio se llevó a cabo utilizando un diseño cuasi-experimental de un solo grupo con pruebas pre-test y post-test, y debido a la naturaleza de la intervención, no se aplicó ningún tipo de enmascaramiento ni a las adolescentes ni a los investigadores. El estudio utilizó la declaración TREND (Transparent Reporting of Evaluations with Nonrandomized Designs), que permite la presentación transparente de informes de estudios controlados no aleatorios para establecer una base empírica para la toma de decisiones relacionadas con la salud pública^(16)^.

### Cálculo del Tamaño de la Muestra

La población del estudio estuvo compuesta por las 284 adolescentes matriculadas en los grados 6.º y 7.º de una escuela secundaria del centro de la ciudad de Ankara, durante el semestre de primavera del año académico 2023-2024. Dado que en la literatura se indicaba que la edad media de la menarca en Turquía era de 12-13 años, la población estuvo compuesta por alumnas de 6.º y 7.º grado^(17)^. El tamaño de la muestra se calculó en 199 estudiantes con una potencia del 80%, una probabilidad de error alfa de 0,05 y un tamaño del efecto pequeño (Cohen d = 0,2) utilizando una distribución bilateral. El estudio se completó con 267 adolescentes, alcanzando una potencia del 90%. En los diseños de pre-test y post-test de un solo grupo, se suele recomendar un tamaño del efecto de 0,2 para detectar cambios pequeños pero significativos; por lo tanto, se eligió este límite para el cálculo del tamaño de la muestra^(18)^. Se utilizó el programa G Power 3.1.9.4 para calcular el tamaño de la muestra. Se utilizó un método de muestreo por conveniencia para realizar el estudio. En el momento del estudio, se excluyó de la muestra a 10 adolescentes que informaron haber recibido previamente educación sobre la menstruación por parte de sus maestros y a 7 adolescentes que no asistieron a la educación durante el estudio. La muestra estuvo compuesta por 267 adolescentes que estaban presentes en la escuela durante la recolección de datos y que respondieron los formularios de recolección de datos antes de la educación (AE) y un mes después de la educación (DE). Se preparó un flujograma basado en la lista de verificación TREND^(16)^ y la Figura 1 muestra el flujograma del estudio.

**Figura 1:** Diagrama de flujo del estudio.

### Criterios de Inclusión y Exclusión

Los criterios de inclusión fueron los siguientes: tener características biológicas femeninas; tener entre 11 y 14 años; estar matriculada en el sexto o séptimo grado de la escuela donde se imparte la educación; estar en la escuela el día del estudio; no tener barreras de comunicación; no tener barreras para responder a las preguntas de la prueba previa y del post-test; participar voluntariamente en la educación.

Los criterios de exclusión fueron los siguientes: no poder participar en la educación debido a necesidades educativas especiales; no participar en la educación que se impartirá en el marco de la investigación; haber recibido previamente educación formal sobre la menstruación.

### Herramientas para la Recolección de Datos

Los datos de la investigación se recolectaron de adolescentes matriculadas en 6.º y 7.º grado en una escuela secundaria situada en el centro de la ciudad de Ankara durante el semestre de primavera del año académico 2023-2024, utilizando un «formulario sociodemográfico» y un «formulario de prueba de conocimientos» administrados mediante entrevistas presenciales.

El formulario sociodemográfico creado por los investigadores consta de un total de nueve preguntas: seis preguntas que abarcan características sociodemográficas como la edad de la adolescente, el nivel de estudios, el nivel educativo de la madre, el nivel educativo del padre, la situación laboral de la madre y la situación laboral del padre; y tres preguntas que abarcan características menstruales, como la menarca, el conocimiento de la información relacionada con la menstruación y la fuente de información recibida sobre la menstruación^(1,11)^.

El formulario de la prueba de conocimientos constaba de 10 preguntas sobre el período menstrual y las prácticas de higiene relacionadas con la menstruación. Además, el formulario contenía preguntas sobre el nombre, los apellidos y el nivel de estudios de las adolescentes. Las preguntas tenían las opciones «verdadero», «falso» y «no sé». Para evaluar la validez del contenido de una escala, se recomienda obtener la opinión de entre 3 y 20 expertos sobre la idoneidad de los ítems de la escala^(19)^. Por lo tanto, se solicitó la opinión de expertos al consejero escolar y a tres académicos para garantizar la relevancia y la idoneidad de las preguntas de la prueba de conocimientos. El índice de validez del contenido de cada pregunta del formulario, calculado mediante el método de Davis, osciló entre 0,8 y 1,0^(20)^. La prueba de conocimientos sobre la menstruación se utilizó tanto para la recolección de datos pre-test como para la recolección de datos post-test. Para evaluar la comprensibilidad de los formularios de recolección de datos, se realizó una aplicación previa con 10 adolescentes de la muestra. Tras la aplicación previa, se finalizaron los formularios de recolección de datos. La consistencia interna de la prueba de conocimientos se examinó utilizando la fórmula 20 de Kuder-Richardson (KR-20). Para la administración de la prueba previa, el coeficiente KR-20 se calculó en 0,48, lo que indica una consistencia interna relativamente baja. En el post-test, el coeficiente KR-20 disminuyó aún más hasta 0,26. Esta disminución puede explicarse por la reducción de la varianza de las puntuaciones totales en el post-test, ya que la mayoría de las adolescentes obtuvieron puntuaciones muy altas (M = 9,63, DE = 0,88). Estos resultados sugieren que, si bien la prueba fue útil para detectar diferencias en los conocimientos iniciales, se volvió menos discriminatoria después de la intervención, cuando los niveles de conocimiento de las adolescentes eran homogéneamente altos.

### Recolección de Datos

Antes de la intervención, se evaluaron los conocimientos iniciales de las adolescentes sobre la menstruación. El programa educativo tenía como objetivo mejorar su comprensión e incluía una presentación en PowerPoint que abarcaba la anatomía de los órganos genitales internos y externos femeninos, la fisiología de la menstruación, la higiene menstrual y perineal, los mitos comunes sobre la menstruación y el apoyo psicosocial. Las sesiones se impartieron en persona utilizando técnicas interactivas, como preguntas y respuestas, demostraciones y debates en grupo. Durante las demostraciones, los investigadores explicaron y modelaron primero las prácticas de higiene menstrual, tras lo cual las adolescentes practicaron estas actividades por sí mismas. Para fomentar la participación, las respuestas correctas durante los segmentos de preguntas y respuestas se recompensaron con bolígrafos. Las adolescentes se organizaron en grupos de aproximadamente 50 chicas por sesión, y todas las sesiones se llevaron a cabo en la sala de conferencias de la escuela. La intervención constó de dos sesiones de aproximadamente 40 minutos, lo que supuso una duración total de 80 minutos. El proceso del estudio comenzó con la recolección de los datos sociodemográficos de las adolescentes, seguida de la administración de un cuestionario de conocimientos como prueba previa. A continuación, se llevaron a cabo las sesiones educativas y, un mes después de la intervención, se volvió a administrar el mismo cuestionario de conocimientos como prueba post-test. Antes de las sesiones, se informó a las adolescentes sobre el propósito de la educación, los formularios que debían completar y cómo hacerlo, lo que, junto con los incentivos interactivos, sirvió para mejorar la adherencia y la participación.

## RESULTADOS

El resultado principal del estudio fue el cambio en la puntuación total de conocimientos sobre la menstruación de las adolescentes antes y después de la educación. El resultado secundario fue la diferencia en los niveles de conocimiento después de la intervención según el estado de la menarca de las adolescentes.

### Análisis y Tratamiento de Datos

Los datos del estudio se analizaron con el paquete estadístico para las ciencias sociales (SPSS) versión 25,0. Los datos categóricos se expresaron como porcentaje, media y desviación estándar. La distribución normal de los datos se comprobó con Kolmogorov-Smirnov. Se utilizaron la prueba t para muestras emparejadas y el análisis de correlación de Pearson para comparar las puntuaciones previas y el post-test de la prueba de conocimientos. Se realizó un análisis de regresión lineal múltiple para determinar los factores que afectan al nivel de conocimientos sobre la menstruación. Se utilizó el coeficiente d de Cohen para calcular el tamaño del efecto^(18)^. Se aceptó un nivel de significación estadística de 0,05. Se utilizó el análisis de varianza unidireccional (ANOVA) para comparar las puntuaciones de tres o más grupos, y la prueba post hoc de Bonferroni para determinar el grupo diferente. Se utilizó un análisis de regresión lineal múltiple para determinar la relación entre las variables dependientes e independientes. Una vez cumplida la hipótesis de normalidad para el análisis, se añadieron las siguientes variables utilizando el método escalonado: edad de la estudiante, grado de educación, nivel educativo y situación laboral de la madre y el padre, estado de la menarca, recepción de información sobre la menstruación y fuente de información sobre la menstruación. Se cumplieron las condiciones del análisis de regresión al determinar que el modelo no presentaba problemas de multicolinealidad (factor de inflación de la varianza: 1,004) ni de autocorrelación (Durbin-Watson: 1,857) y que los residuos estándar se encontraban entre (-3+3) con un intervalo de confianza del 95%^(21)^.

### Aspectos Éticos

Se obtuvo el permiso para la investigación del Comité de Ética en Investigación Científica y Publicaciones de Ciencias Sociales y Humanas de la Universidad de Başkent (Fecha: 6/11/2023-Número: 17162298.600-241). Tras la aprobación del comité de ética, también se obtuvo el permiso institucional de la escuela donde se llevaría a cabo el estudio. Se incluyó una declaración escrita en la que se explicaba el propósito del estudio en la introducción del formulario de recolección de datos, y se incluyó en el estudio a las adolescentes que aceptaron participar en él después de leerla. Además, el conjunto de datos que contenía las respuestas rellenadas por las adolescentes se guardó en un archivo cifrado en la computadora y, una vez finalizado el análisis de los datos, estos se eliminaron para garantizar la confidencialidad de los datos personales. El estudio se llevó a cabo de conformidad con la «Declaración de Helsinki».

## RESULTADOS

La comparación de las puntuaciones de conocimiento de las adolescentes sobre la menstruación antes y después del post-test, según las características sociodemográficas, se presenta en la Tabla 1. La edad media de las adolescentes era de 12,38 ± 0,7 años. De las adolescentes incluidas en el estudio, el 44,6% tenía 13 años y el 68,5% estaba matriculado en séptimo grado. En cuanto a la educación de los padres, el 32,6% de las madres había completado la escuela primaria, mientras que el 40,4% de los padres era graduado de secundaria. La mayoría de las madres no tenían empleo (77,2%), mientras que la mayoría de los padres sí lo tenían (93,6%). Además, el 61,8% de las adolescentes informaron haber tenido la menarca y, entre las que habían recibido información sobre la menstruación, la principal fuente de información era su madre (71,1%) (Tabla 1).

**Tabla 1:** Distribución de las puntuaciones medias de conocimiento previas y post-test de las adolescentes según sus características sociodemográficas y menstruales – Ankara, Turquía, 2023.

Características (n:267)		n	%	Puntuación de pre test	Puntuación de post test
				Media ± DE	Media ± DE
**Edad** [X̄: 12,38; DE: 0,7 years]	11	32	12,0	6,03 ± 1,7	8,87 ± 1,1
	12	107	40,1	5,97 ± 2,1	9,26 ± 1,2
	13	119	44,6	6,76 ± 1,7	9,14 ± 1,3
	14	9	3,4	6,55 ± 1,7	9,00 ± 0,9
Test				^†^3,798; ^*^0,005^a^	^†^0,798; ^*^0,527
**Puntuación**	6	84	31,5	6,01 ± 2,0	9,04 ± 1,3
	7	183	68,5	6,53 ± 1,9	9,20 ± 1,2
Test				^‡^–2,085; ^*^0,038	^‡^–1,006; ^*^0,315
**Nivel educativo de la madre**	Analfabeta	5	1,9	4,80 ± 2,2	7,20 ± 2,9
	Primaria	87	32,6	6,06 ± 1,9	9,21 ± 1,1
	Secundaria	78	29,2	6,33 ± 1,8	9,01 ± 1,3
	Post secundaria	82	30,7	6,68 ± 1,9	9,30 ± 1,1
	Universidaad	15	5,6	7,06 ± 1,5	9,40 ± 0,8
Test				^†^2,529; ^*^0,041 ^b^	^†^4,283; ^*^0,002 ^b^
**Nivel educacional del padre**	Analfabeto	5	1,9	5,40 ± 2,4	9,40 ± 0,9
	Primaria	44	16,5	6,00 ± 1,9	9,22 ± 0,9
	Secundaria	81	30,3	6,17 ± 1,8	9,03 ± 1,5
	Post secundaria	108	40,4	6,69 ± 1,9	9,15 ± 1,2
	Universidaad	29	10,9	6,41 ± 2,1	9,34 ± 0,7
Test				^†^1,777; ^*^0,134	^†^0,460; ^*^0,765
**Empleo de la madre**	Empleada	61	22,8	6,40 ± 1,8	9,09 ± 1,3
	No empleada	206	77,2	6,35 ± 1,9	9,17 ± 1,2
Test				^‡^0,200; ^*^0,842	^‡^ –0,434; ^*^ 0,665
**Empleo del padre**	Empleado	250	93,6	6,38 ± 1,9	9,18 ± 1,2
	No empleado	17	6,4	6,05 ± 1,7	8,70 ± 1,7
Test				^‡^0,691; ^*^0,490	^‡^1,599; ^*^0,111
**Menarca**	Si	165	61,8	6,78 ± 1,7	9,22 ± 0,1
	No	102	38,2	5,69 ± 2,0	9,04 ± 0,1
Test				^‡^4,795; ^*^0,001	^‡^1,153; ^*^0,250
**Información sobre menstruacion**	Madre	165	71,1	6,60 ± 1,75	9,20 ± 1,20
	Amiga o hermana	65	28,9	6,16 ± 1,84	8,98 ± 1,34
Test				^‡^1,678; ^*^0,095	^‡^1,185; ^*^0,237
**Recibió información sobre mensatruación**	Si	230	86,1	6,50 ± 1,8	9,20 ± 1,2
	No	37	13,9	5,48 ± 2,5	8,94 ± 1,2
Test				^‡^2,433; ^*^0,019	^‡^0,925; ^*^0,252

^a^Según la prueba de Bonferroni, la diferencia se da entre los 12 y los 13 años;

^b^Según la prueba de Bonferroni, la diferencia se debe al grupo con madres analfabetas;

^†^: Análisis de varianza unidireccional;

^‡^: Prueba t de Student; DE: Desviación estándar; X: Media;

^*^p < 0,05.

Se observó una diferencia significativa en las puntuaciones de conocimientos previos a la prueba según la edad (p = 0,005), ya que las niñas de 12 años obtuvieron puntuaciones medias más bajas que las de 13 años (5,97 ± 2,1 frente a 6,76 ± 1,7, respectivamente).

Del mismo modo, las alumnas de séptimo grado obtuvieron puntuaciones significativamente más altas en la prueba previa en comparación con las alumnas de sexto grado (p = 0,038).

Se observó que el nivel de educación de la madre estaba significativamente asociado con las puntuaciones obtenidas tanto en la prueba previa (p = 0,041) como en la prueba post-test (p = 0,002). Las adolescentes cuyas madres eran analfabetas obtuvieron puntuaciones medias más bajas en comparación con aquellas cuyas madres tenían algún nivel de educación formal. Las puntuaciones medias en la prueba previa y post-test fueron de 4,80 ± 2,2 y 7,20 ± 2,9 para las niñas con madres analfabetas; 6,06 ± 1,9 y 9,21 ± 1,1 para aquellas cuyas madres habían terminado la primaria; 6,33 ± 1,8 y 9,01 ± 1,3 para las que tenían estudios secundarios; 6,68 ± 1,9 y 9,30 ± 1,1 para las que tenían estudios de bachillerato; y 7,06 ± 1,5 y 9,40 ± 0,8 para las que tenían estudios universitarios, respectivamente. Por el contrario, el nivel educativo del padre y la situación laboral de la madre y el padre no se asociaron con diferencias significativas en las puntuaciones de conocimiento previas y post-test (p > 0,05) (Tabla 1).

La comparación de las puntuaciones de conocimientos según las características relacionadas con la menstruación también se muestra en la Tabla 1. Las puntuaciones de conocimientos previas a la prueba fueron significativamente más altas entre las niñas que habían experimentado la menarca en comparación con las que no (p = 0,001), mientras que las puntuaciones del post-test no difirieron significativamente entre los dos grupos (p = 0,250). Del mismo modo, las adolescentes que habían recibido previamente información sobre la menstruación obtuvieron puntuaciones de conocimiento previas a la prueba más altas que las que no la habían recibido (p = 0,019); sin embargo, las puntuaciones de conocimiento post-test fueron comparables entre los grupos (p = 0,252).

La Tabla 2 presenta la comparación general de las puntuaciones de conocimientos previas y post-test. La puntuación media de conocimientos previa a la prueba de todos los participantes fue de 7,16 ± 1,8, que aumentó a 9,62 ± 0,8 después de la intervención. Este aumento fue estadísticamente significativo (p = 0,001), con un tamaño del efecto d de Cohen de 1,3, lo que indica un efecto fuerte. Cuando se analizaron por nivel de grado, las puntuaciones medias previas y de post-test fueron de 6,85 ± 1,9 y 9,54 ± 0,7 para los alumnos de sexto grado, y de 7,30 ± 1,7 y 9,66 ± 0,9 para los alumnos de séptimo grado, respectivamente. Los valores d de Cohen correspondientes fueron de 1,7 para los alumnos de sexto grado y de 1,4 para los de séptimo grado, lo que refleja tamaños del efecto fuertes (Tabla 2).

**Tabla 2:** Comparación de las puntuaciones medias de conocimientos en la prueba previa y la prueba post-test de las adolescentes – Ankara, Turquía, 2023.

Tests	n	X̄– ± DE	Correlación		t test de muestras pareadas			d de Cohen
			r	p	t	DE	p	
**Pre test**	267	7,16 ± 1,84	^§^0,16	^*^0,007	^||^–21,08	266	^*^0,001	1,35
**Post test**	267	9,62 ± 0,87						
**Pre test 6^o^grado**	84	6,85 ± 1,98	^§^0,12	^*^0,275	^||^–12,04	83	^*^0,001	1,78
**Post test 6^o^ grado**	84	9,54 ± 0,78						
**Pre test 7** ^o^ **grado**	183	7,30 ± 1,76	^§^0,17	^*^0,017	^||^–17,35	182	^*^0,001	1,40
**Post test 7** ^o^ **grado**	183	9,66 ± 0,91						

^§^: Coeficiente de correlación de Pearson;

^||^: Prueba t para muestras emparejadas; DE: Desviación estándar; X̄: Media;

^*^p < 0,05.

El análisis de regresión lineal múltiple realizado para determinar las variables independientes que afectan al nivel de conocimiento sobre la menstruación se muestra en la Tabla 3. El análisis se realizó para la puntuación media de conocimiento de la prueba previa. El estado de la menarca y el nivel de educación materna surgieron como predictores estadísticamente significativos (p = 0,001). En conjunto, estas variables explicaron el 11% de la varianza en las puntuaciones de conocimiento de la prueba previa, y se observó una correlación positiva moderada entre estos factores y las puntuaciones de conocimiento de la prueba previa (r = 0,33).

**Tabla 3:** Análisis de regresión lineal múltiple de las variables que afectan al nivel de conocimiento sobre la menstruación – Ankara, Turquía, 2023.

Modelo	b	SE	β	t (p)	R	R^2^
**Menarca**	–1,062	0,227	–0,272	–4,674 (**0,001**)	0,33	0,11
**Nivel educativo de la madre**	0,324	0,115	0,165	2,825 (0,005)		

β: Coeficientes beta estandarizados; b: Coeficiente de regresión no estandarizado; R: Coeficiente de correlación múltiple; SE: Error estândar.

## DISCUSIÓN

Este estudio examinó los conocimientos y prácticas de las adolescentes en relación con la menstruación y evaluó la eficacia de una intervención educativa estructurada. Aunque la edad de la menarca varía en función de factores biológicos, ambientales y socioculturales, generalmente se produce entre los 11 y los 16 años^(1)^, con una edad media de 13 años según la bibliografía^(17,22)^. En el presente estudio, las adolescentes de séptimo grado, la mayoría de las cuales tenían 13 años, demostraron puntuaciones más altas en los conocimientos previos sobre la menstruación en comparación con otros grupos de edad. Es probable que este hallazgo esté asociado con una mayor conciencia, una experiencia personal temprana con la menstruación^(23)^ y un mayor interés en las cuestiones menstruales durante este periodo de desarrollo^(24)^.

Estudios anteriores han informado de que los conocimientos de las adolescentes sobre la gestión de la higiene menstrual suelen ser insuficientes y estar influenciados por tabúes culturales^(14)^. Sin embargo, algunos estudios indican que una proporción considerable de niñas posee conocimientos suficientes sobre la menstruación antes de la menarca^(6)^. En el presente estudio, los niveles de conocimiento más altos en la prueba previa entre las niñas de 13 años y las que ya habían experimentado la menarca pueden explicarse por la experiencia personal y el aprendizaje informal a través del entorno familiar y social. Las diferencias observadas entre los estudios pueden reflejar variaciones en las normas culturales, el estatus socioeconómico y el acceso a información precisa.

La menarca suele producirse durante el séptimo grado, y en este estudio, las puntuaciones de conocimiento post-test de las alumnas de séptimo grado fueron más altas que las de las alumnas de sexto grado. Estos hallazgos respaldan la eficacia de la educación sobre higiene menstrual. Se han obtenido resultados similares en estudios anteriores, que muestran cambios positivos en los hábitos de aseo y los comportamientos de higiene menstrual tras la educación sobre higiene genital^(25,26)^. Sinop Gedik y Şahin^(27)^ también demostraron que la educación sobre higiene menstrual mejoraba significativamente los conocimientos y los comportamientos de higiene de las adolescentes. En consonancia con estos hallazgos, el aumento de las puntuaciones del post-test observado en este estudio sugiere una reducción de los conceptos erróneos y un aumento de la concienciación, lo que lleva a la aceptación de la hipótesis H1. En general, la intervención educativa fue eficaz para mejorar los conocimientos menstruales de las adolescentes.

Una información precisa y fiable sobre la menstruación es esencial para mantener una higiene adecuada y gestionar la menstruación de forma eficaz. La difusión de información sobre la salud menstrual ha cobrado cada vez más importancia, especialmente en los países en desarrollo^(28)^. Los estudios indican que los conocimientos menstruales de las niñas suelen ser insuficientes y se ven influidos negativamente por las creencias culturales, mientras que la comunicación con las madres sobre la menstruación puede ser limitada^(11)^. La literatura identifica sistemáticamente a las madres como la principal fuente de información menstrual para las niñas^(6,28)^, y destaca que el nivel educativo de las madres desempeña un papel fundamental para garantizar un conocimiento preciso y unas prácticas de higiene saludables^(15)^.

En el presente estudio, la mayoría de las adolescentes identificaron a sus madres como su principal fuente de información menstrual, y los niveles más altos de educación materna se asociaron con un mayor conocimiento menstrual entre las hijas. Además, las niñas cuyas madres eran analfabetas obtuvieron puntuaciones más bajas en los conocimientos previos a la prueba, lo que pone de relieve la influencia de la educación materna en la conciencia menstrual. Estos hallazgos coinciden con los comunicados por Siabani, Charehjow y Babakhani^(29)^, quienes encontraron una relación significativa entre los niveles de educación de las madres y las prácticas menstruales de las adolescentes. El conocimiento materno inadecuado puede dificultar la comunicación eficaz entre madres e hijas sobre la salud menstrual^(11)^, mientras que la mejora del nivel educativo de las madres puede aumentar la precisión de la información que comparten con sus hijas^(30)^.

El conocimiento sobre la menstruación antes de la menarca se reconoce como un indicador clave para monitorear la gestión de la higiene menstrual tanto a nivel nacional como mundial^(11,23)^. En este estudio, las adolescentes que habían experimentado la menarca y habían recibido información sobre la menstruación obtuvieron puntuaciones más altas en los conocimientos previos a la educación. Mientras que algunos estudios informan de que las niñas premenárquicas tienen un conocimiento limitado de la menstruación y desconocen el origen del sangrado menstrual^(31)^, otros indican que las niñas que experimentan la menarca adquieren mayores conocimientos, confianza y competencia en el manejo de la menstruación^(23)^. Aunque Gümüş Sarı^(32)^ informó de puntuaciones más bajas en los conocimientos previos a la educación entre las niñas que habían experimentado la menarca, esta diferencia no fue estadísticamente significativa. Los niveles de conocimiento más altos observados entre las adolescentes posmenarca en el presente estudio probablemente se deban a la experiencia personal y al aprendizaje ambiental, mientras que las discrepancias entre los estudios pueden deberse a diferencias culturales, socioeconómicas e informativas.

## LIMITACIONES

Las limitaciones de este estudio incluyen la inclusión de adolescentes con y sin menarca en el grupo de muestra, la falta de pruebas suficientes de validez y fiabilidad del cuestionario administrado a las adolescentes antes y después de la intervención educativa, y el alcance limitado de la consulta a expertos durante el desarrollo de las herramientas de recolección de datos. En particular, la fiabilidad relativamente baja de la prueba de conocimientos utilizada en el estudio constituye una clara limitación metodológica y debe tenerse en cuenta al interpretar los resultados.

Además, aunque se incluyó un número suficiente de adolescentes en este estudio, es importante realizar estudios similares en diferentes regiones para respaldar un conocimiento preciso del ciclo menstrual, ya que la información y las percepciones relacionadas con la menstruación pueden verse influidas por las diferencias culturales y ambientales.

## CONCLUSIÓN

En nuestro estudio, se observó que la educación era eficaz y que las puntuaciones de las pruebas de conocimientos de las adolescentes sobre la menstruación aumentaron después de la educación. Se observó que la menarca y el nivel educativo de la madre eran factores que influían en el nivel de conocimientos sobre la menstruación. Por lo tanto, se postula que el aumento del nivel educativo de las madres tendrá un efecto positivo, aumentando las prácticas correctas relacionadas con la menstruación antes de la menarca en las adolescentes.

Los hallazgos relacionados con los factores que afectan a la higiene menstrual de las adolescentes pueden orientar las políticas. Los responsables políticos pueden utilizar esta información para garantizar que la educación sobre higiene menstrual se imparta regularmente en todas las escuelas y se incluya en el plan de estudios. Se recomienda que este programa educativo se lleve a cabo en colaboración con personal sanitario especializado y que se tengan en cuenta la edad y la cultura de las adolescentes, así como los mitos que existen en sus comunidades, a la hora de planificar la educación. Además, este estudio puede orientar futuras investigaciones para estudiar la eficacia de estas intervenciones e identificar otras deficiencias en la higiene menstrual que puedan dar lugar a mejoras específicas.

Educar a las adolescentes sobre la higiene menstrual es esencial para promover la salud escolar, ya que les proporciona los conocimientos y las habilidades necesarios para manejar la menstruación de forma segura e higiénica. Una educación adecuada sobre la higiene menstrual ayuda a prevenir problemas de salud, como las infecciones, al fomentar el uso de productos menstruales adecuados. Además, desempeña un papel crucial en la reducción del estigma y la vergüenza que a menudo se asocian con la menstruación, fomentando así un entorno de apoyo e inclusión. Al incorporar la educación sobre higiene menstrual en los planes de estudio escolares, se puede reducir el absentismo, lo que garantiza la participación continua en la educación y mejora el bienestar general de las adolescentes.

## Data Availability

El conjunto de datos que respaldan las conclusiones de este estudio está disponible previa solicitud al autor correspondiente.
